# Normoferritinemic Versus Hyperferritinemic Inflammation in Patients Admitted to the Department of Internal Medicine

**DOI:** 10.3390/jcm15072646

**Published:** 2026-03-31

**Authors:** Saar Beit Yaakov, Ori Argov, Ori Rogowski, Chen Klechevski, Saritte Perlman, Moshe Shtark, Tomer Ziv Baran, Shlomo Berliner, Asaf Wasserman

**Affiliations:** 1 Internal Medicine “C” Department, Tel Aviv Sourasky University Medical Center, Tel Aviv 6423906, Israel; 2 Gray Faculty of Medical & Health Sciences, Tel Aviv University, Tel Aviv 6997801, Israel; 3 Department of Health Policy and Management, Faculty of Health Sciences, Ben Gurion University of the Negev, Beersheva 8410501, Israel; 4 Division of clinical Laboratories, Tel-Aviv Sourasky University Medical Center, Tel Aviv 6423906, Israel; 5 Department of Epidemiology and Preventive Medicine, School of Public Health, Gray Faculty of Medical & Health Science, Tel Aviv University, Tel Aviv 6139001, Israel; 6 Internal Medicine “E” Department, Tel Aviv Sourasky University Medical Center, Tel Aviv 6423906, Israel

**Keywords:** hyperferritinemia, normoferritinemia, inflammation, C-reactive protein, sepsis, prognostic biomarker, macrophage activation

## Abstract

**Background**: Screening patients admitted to internal medicine for hyperferritinemia might reveal a dichotomy between normoferritinemic inflammation and hyperferritinemic inflammation phenotypes, opening new research into innate immunity activation during acute inflammation. **Methods**: We identified 4514 consecutive patients screened for CRP and ferritin on admission. Patients with CRP ≤ 150 mg/L were excluded. We selected 100 patients with the lowest (normoferritinemic inflammation) and 100 with the highest (hyperferritinemic inflammation) ferritin concentrations. Sub-analysis of 39 CRP-matched pairs (±15 mg/L) and multivariable logistic regression—adjusting for age, sex, sepsis, malignancy, CRP, and comorbidities—were performed. **Results**: Groups did not differ significantly by age (*p* = 0.068) or sex (*p* = 0.319). Mortality was significantly higher in the hyperferritinemic inflammation group (41% vs. 7%, *p* < 0.001), a trend maintained in non-malignant (31.1% vs. 6.5%, *p* < 0.001) and CRP-matched (25.6% vs. 2.6%, *p* = 0.012) subgroups. Multivariable regression confirmed hyperferritinemic inflammation as a significant independent predictor of mortality (OR 3.726; 95% CI 1.304–10.647; *p* = 0.014), even after adjusting for the Charlson Comorbidity Index. **Conclusions**: Significant inflammation accompanied by hyperferritinemic inflammation is associated with elevated mortality compared to normoferritinemic inflammation, suggesting a dichotomous divergence of the inflammatory response.

## 1. Introduction

There are multiple lines of evidence to suggest that hyperferritinemia is associated with significant diseases/disorders and worse prognosis [1,2,3,4,5,6,7,8,9,10,11]. Although reported to be part of the acute phase response [12], it is not entirely clear whether all inflammatory responses are necessarily associated with hyperferritinemia or whether hyperferritinemia is a more specific response in certain inflammatory phenotypes [13]. Indeed, our previous human model of acute bacterial infections identified a specific patient phenotype. These individuals exhibited a significant inflammatory response without concomitant hyperferritinemia [14]. Similar findings were observed in a canine model [15]. We have now taken advantage of the fact that one of our departments of Internal Medicine is screening all admitted patients for both C-Reactive Protein (CRP) and Ferritin.

We focused on a cohort of patients with significant systemic inflammation, defined by CRP levels exceeding 150 mg/L. By comparing individuals with extremely low versus extremely high ferritin concentrations, we aimed to evaluate the prognostic significance of this divergence. We hypothesized that hyperferritinemic inflammation identifies a specific clinical subgroup with higher in-hospital mortality, potentially reflecting a distinct inflammatory phenotype compared to those with normoferritinemic inflammation.

## 2. Materials and Methods

### 2.1. Study Design, Setting and Population

This is an exploratory, retrospective cohort study of all patients admitted to the Department of Internal Medicine ‘C’ at the Tel Aviv Sourasky Medical Center between May 2021 and March 2025. In this department, all patients are routinely screened for both C-reactive protein (CRP) and ferritin within the first 24 h of admission. A portion of this cohort formed the basis of our previous report regarding the prognostic significance of low-grade ferritinemia [16]. For the present study, only patients with significant inflammation (CRP > 150 mg/L) were included. From this population, we selected the 100 patients with the lowest ferritin concentrations (normoferritinemic-inflammation group) and the 100 patients with the highest ferritin concentrations (hyperferritinemic-inflammation group). This exploratory extreme-groups design was employed to evaluate whether patients with normoferritinemic versus hyperferritinemic inflammation represent two distinct clinical phenotypes. We reasoned that if no significant differences were detected between these extreme groups, the intermediate population would likely not warrant further investigation; therefore, patients with intermediate ferritin values were not included in the present study. Two sub-analyses were performed. In the first sub-analysis, only patients without active malignancy (active oncologic treatment and/or stage IV disease) were included. In the second sub-analysis, patients were paired according to their CRP values, ±15 mg/L was used as a criterion for matching.

### 2.2. Study Variables

Data for this study were retrieved through a combined approach, utilizing both the direct review of electronic medical records and the “MDClone” platform at the Tel-Aviv Sourasky University Medical Center (https://mdclone.com, Beer-sheva, Israel) [17]. The variables extracted for each patient included demographic information (age and sex), a comprehensive profile of pre-existing comorbidities, and the primary diagnosis upon admission. Additionally, the dataset incorporated laboratory values for ferritin and CRP concentrations, alongside clinical outcomes defined by in-hospital mortality and the specific documented cause of death.

### 2.3. Laboratory Methods

The concentration of a wide range of C-reactive protein (wrCRP) was measured on the ADVIA 2400 Chemistry System using a latex-enhanced immunoturbidimetric assay, with turbidity read at 571 nm. The analytical measuring range was 0.03–164 mg/L; an automatic rerun condition extended the reportable range to 624 mg/L. Calibration was performed with ADVIA Chemistry Wide-Range C-Reactive Protein calibrators at least every 21 days and as indicated by quality-control procedures. The method is traceable to IRMM CRM-470.

Ferritin levels were measured on the ADVIA Centaur system using a two-site sandwich direct chemiluminometric immunoassay. The analytical measuring range was 0–1650 ng/mL. Samples exceeding this analytical range were diluted with a specific diluent and re-analyzed for a result. Calibration was performed using the ADVIA Centaur Calibrator “C” at 28-day intervals, or as required based on Quality Control results. Results are traceable to the WHO 2nd International Standard (80/578) [18].

### 2.4. Statistical Methods

Categorical variables were summarized using frequency and percentage. The distribution of continuous variables was evaluated using a histogram. Since all continuous variables were skewed, they were reported as median and interquartile range (IQR). The Chi-square test and Fisher’s exact test were applied to compare categorical variables between groups, and the Mann–Whitney test was used to compare continuous variables. The Wilcoxon test and McNemar test were used, respectively, to compare continuous and categorical variables between the two matched groups. To identify independent predictors of in-hospital mortality, multivariable logistic regression analyses were performed. The first model included age, sex, sepsis, active malignancy, CRP levels, and the ferritin group (hyperferritinemic versus normoferritinemic). To further account for the impact of underlying comorbidities, a second adjusted model was constructed by adding the Charlson Comorbidity Index (CCI) as a covariate. Results are reported as odds ratios (OR) with corresponding 95% confidence intervals (CI). All statistical tests were two-sided, and *p* < 0.05 was considered statistically significant. Statistical analysis was performed using SPSS software (IBM SPSS Statistics for Windows, version 29, IBM Corp., Armonk, NY, USA, 2023).

## 3. Results

### 3.1. Study Population and Selection Flow

**Figure 1:**
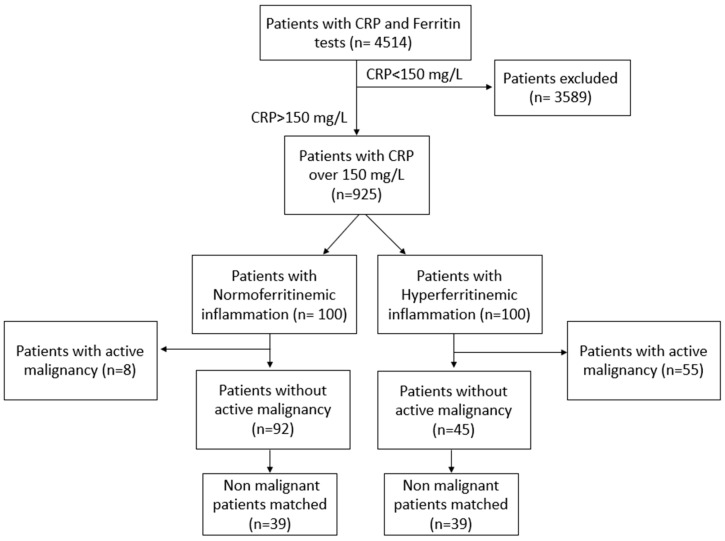
Patient’s Flow chart.

A total of 4514 patients admitted to the Department of Internal Medicine “C” at the Tel-Aviv Sourasky University Medical Center were initially screened, all of whom underwent routine testing for both Ferritin and CRP within the first 24 h of hospitalization. Among this initial cohort, 925 patients were identified as having significant systemic inflammation, defined by CRP concentrations exceeding 150 mg/L. For the comparative analysis, these patients were divided into two distinct groups of 100 patients each, representing the highest and lowest ferritin concentrations within the inflammatory cohort, as detailed in the study flow chart (Figure 1). The baseline characteristics of the two groups, classified as normoferritinemia (*n* = 100) and hyperferritinemia (*n* = 100), are presented in Table 1.

### 3.2. Normoferritinemic-Inflammation Versus Hyperferritinemic-Inflammation Groups

The median ferritin level in the normoferritinemic group was 88.50 ng/mL (IQR 70.05–109.85), while the hyperferritinemic group exhibited a significantly higher median level of 2382.10 ng/mL (IQR 1863.07–4054.00). Although the hyperferritinemic group tended to be younger (median age 69.2 vs. 76.3 years; *p* = 0.068) and showed a higher proportion of males (59% vs. 52%; *p* = 0.319), neither parameter reached statistical significance. In contrast, inflammatory markers were significantly more pronounced in the hyperferritinemic group, with a median CRP of 207.03 mg/L compared to 176.77 mg/L in the normoferritinemic cohort (*p* < 0.001). These laboratory differences were accompanied by distinct clinical profiles, as the distribution of admission diagnoses varied significantly between the two groups (*p* < 0.001); specifically, sepsis was markedly more prevalent in the hyperferritinemic group (28% vs. 5%), whereas urinary tract infections (20% vs. 4%) and cellulitis (17% vs. 5%) were more frequently observed in the normoferritinemic group, while pneumonia remained common in both cohorts (30% vs. 31%). Comorbidity patterns also differed sharply, with active malignancy being substantially more common in patients with hyperferritinemia (55% vs. 8%; *p* < 0.001), while the normoferritinemic group demonstrated higher rates of hypertension (62% vs. 47%; *p* = 0.033), diabetes mellitus (43% vs. 25%; *p* = 0.007), and a history of cerebrovascular accident (24% vs. 2%; *p* < 0.001). Ultimately, these laboratory and clinical disparities culminated in a profound difference in outcomes, with the hyperferritinemic group experiencing a significantly higher rate of in-hospital mortality compared to the normoferritinemic group (41% vs. 7%; *p* < 0.001).

### 3.3. Sub-Analysis of Patients Without Active Malignancy

Given the high prevalence of active malignancy observed in the primary study cohort, an additional sub-analysis was performed to evaluate the clinical significance of ferritin levels independently of oncological influences. This analysis focused on a subgroup of 137 patients without active malignancy, comprising 92 patients with normoferritinemia and 45 patients with hyperferritinemia, as summarized in Table 2. Within this cohort, median ferritin levels were 86.65 ng/mL (IQR 69.92–108.30) in the normoferritinemic group compared to a markedly higher 2135.40 ng/mL (IQR 1715.70–3625.95) in the hyperferritinemic group. While demographic characteristics such as age (*p* = 0.294) and sex (*p* = 0.537) did not differ significantly between the two groups, patients in the hyperferritinemia cohort presented with a more intense systemic inflammatory response, evidenced by significantly higher median CRP levels (220.94 mg/L vs. 174.69 mg/L; *p* < 0.001). The distribution of admission diagnoses also varied considerably (*p* = 0.001), with post hoc analysis revealing that sepsis was notably more prevalent in the hyperferritinemic group (28.9% vs. 5.4%), whereas urinary tract infections were more common in the normoferritinemic cohort (19.6% vs. 6.7%). Regarding baseline comorbidities, patients with normal ferritin levels exhibited a significantly higher prevalence of prior cerebrovascular accidents (23.9% vs. 2.2%; *p* = 0.001) and chronic heart failure (20.7% vs. 6.7%; *p* = 0.036). Despite the absence of active malignancy, in-hospital mortality remained significantly elevated in the hyperferritinemic group compared to the normoferritinemic group (31.1% vs. 6.5%; *p* < 0.001). A detailed assessment of mortality etiologies, as summarized in Table 3, further underscored these clinical disparities; sepsis was identified as the primary cause of death in the hyperferritinemic group, accounting for 50.0% of fatalities, followed by pneumonia (21.4%) and endocarditis (14.2%). In contrast, pneumonia was the leading cause of death among normoferritinemic patients (50.0%), with the remaining deaths attributed to sepsis, HIV, and sudden cardiac death (16.6% each). A systematic analysis of mortality etiologies was not performed for the entire cohort due to the diagnostic complexity inherent in patients with advanced malignancy. In these cases, distinguishing between the acute infectious process and the progression of the underlying terminal disease as the primary cause of death is often clinically ambiguous, potentially confounding the analysis of mortality drivers.

### 3.4. Sub-Analysis of 39 Pairs Without Advanced Malignancy

To further isolate the prognostic impact of ferritin from the potential confounding effects of systemic inflammation intensity, a matched-pair sub-analysis was performed involving 39 pairs of non-active malignant patients. In this analysis, patients with hyperferritinemia were matched with patients with normoferritinemia based specifically on their CRP concentrations to eliminate the influence of inflammation severity on clinical outcomes. Following the matching process, there was no significant difference in median CRP levels between the hyperferritinemic group (219.2 mg/L; IQR 188.0–255.6) and the normoferritinemic group (216.7 mg/L; IQR 190.0–256.5; *p* = 0.675). Demographic variables, including median age (69.3 vs. 77.9 years; *p* = 0.204) and male sex distribution (41% vs. 64%; *p* = 0.064), were also comparable between the two groups. Despite the balanced inflammatory and demographic profiles, in-hospital mortality remained significantly higher in the hyperferritinemic group compared to the normoferritinemic group (25.6% vs. 2.6%; *p* = 0.012). This finding, summarized in Table 4, suggests that hyperferritinemic inflammation serves as an independent predictor of mortality even when the degree of systemic inflammation, as measured by CRP, is held constant.

### 3.5. Independent Predictors of In-Hospital Mortality: Multivariable Logistic Regression Analysis

We performed multivariable logistic regression analyses to evaluate the independent prognostic value of hyperferritinemic inflammation. In the primary model, hyperferritinemia was identified as a significant independent predictor of in-hospital mortality (OR 3.748; 95% CI 1.307–10.744; *p* = 0.014) after adjusting for age, sex, CRP levels, sepsis, and active malignancy. To further account for the potential influence of baseline chronic illness burden, a second model was constructed incorporating the Charlson Comorbidity Index (CCI). The association between hyperferritinemic inflammation and mortality remained stable and statistically significant (OR 3.726; 95% CI 1.304–10.647; *p* = 0.014), while the CCI score itself did not emerge as a significant predictor of mortality in this cohort (*p* = 0.624). These findings demonstrate that extreme hyperferritinemic inflammation identifies a high-risk clinical phenotype, serving as a prognostic marker independent of the patient’s underlying comorbidity burden.

## 4. Discussion

To the best of our knowledge, this is the first study to systematically explore the dichotomy between two distinct inflammatory clinical phenotypes. These include one with concomitant hyperferritinemia and another without a significant increase in ferritin concentrations. The documentation of this dichotomy might pave the way to further explore the hyperferritinemic response as a more specific activation of the innate immune system in certain diseases/disorders rather than a nonspecific biomarker of the acute phase response.

Most patients in the present study presented with acute infections and this is probably related to the fact that these were patients with very high CRP concentrations. Our cohort includes a high number of patients with sepsis and it has already been reported that hyperferritinemic sepsis has a worse prognosis [2,19,20,21]. The present study does confirm these previously reported observations. Indeed, we observed significantly more patients with a diagnosis of sepsis in the hyperferritinemic group, and sepsis was a leading cause of mortality in this group. Ultimately, it might be that hyperferritinemic inflammation may mark a complex of high severity phenotypes, including sepsis, malignancy and/or organ dysfunction.

A relatively large number of patients in the hyperferritinemic group suffered from advanced malignancies. It is not known whether the hyperferritinemia was part of their malignant disease, secondary to their infectious condition or related to both of them. Needless to emphasize that this should be a relevant research question due to the possibility that ferritin might be a pro-inflammatory protein and not necessarily an innocent bystander in the inflammatory response to infection. Infections are leading causes of morbidity and mortality in patients with malignancies and therefore of special research interest. Beyond malignancy and sepsis, it is important to acknowledge that other baseline characteristics—including cerebrovascular disease, diabetes mellitus, and hypertension—also differed significantly between the study groups. These disparities represent potential confounding factors that could have further influenced the observed differences in mortality outcomes. However, our multivariable analysis confirmed that the association between hyperferritinemic inflammation and in-hospital mortality remains robust and independent of these baseline comorbidities.

In order to try and clarify the mutual relations between malignancies, hyperferritinemia and infections, we report the results of our study in a sub-group of patients without advanced malignancy and show that the increased mortality rate in the hyperferritinemic group is similar to the findings in the entire cohort that is composed of both patients with and without advanced malignancy. To further isolate the prognostic impact of ferritin from the intensity of the systemic inflammation, we performed a matched-pair analysis of 39 patients with comparable CRP concentrations (±15 mg/L). This comparison supports the hypothesis that hyperferritinemic inflammation conveys additional prognostic information regarding mortality that is not fully captured by the inflammatory response as measured by CRP. However, we acknowledge that the relatively small sample size of these matched pairs limits the statistical power and robustness of this sub-analysis, and these results should therefore be interpreted as exploratory. One of the main clinical implications of taking into consideration the eventual dichotomy between CRP and Ferritin is the acknowledgement that these two readily available two biomarkers do represent two clinical phenotypes of inflammation namely the IL-1, IL-6 and CRP versus the IL-18 activated Macrophages Ferritin pathways [22,23]. If this is the case, one could raise the possibility that these different phenotypes might also be subjected to different anti-inflammatory therapeutic approaches. Thus, these observations are not necessarily of theoretical relevance.

Beyond its role as a biomarker aiding in the classification of hyperinflammatory states and identifying clinical phenotypes for tailored immunotherapy, emerging evidence suggests that ferritin may also function as a direct mechanistic driver of inflammation. Specifically, ferritin acts as a ligand for the Msr1 receptor, triggering the formation of neutrophil extracellular traps (NETs) that contribute to cytokine storms and multi-organ damage. While our findings highlight a strong association between hyperferritinemic inflammation and in-hospital mortality, the precise pathophysiological contribution and the specific extent to which ferritin actively drives the inflammatory response within our patient cohort remain to be further characterized in future studies [24,25].

The term normoferritinemia might not be accurate for all the patients included here due to the notion that the “true” ferritin concentration might be at times less than 100. In fact, due to the presence of metabolic hyperferritinemia [26] the possibility exists that values that are close to 100 represent, at least in part, the effect of the patients’ dysmetabolic condition and that without this condition the “true” normal ferritin concentration might have been lower. Despite this confounder, we do believe that our term “Normoferritinemic inflammation” is a reasonable expression of the significant discrepancy that can be at times detected in several patients with significant inflammation and no major changes in the concentrations of ferritin.

This study has several limitations that should be acknowledged. First, its retrospective, single-center design may limit the generalizability of the findings. Second, the use of an extreme-groups design, while maximizing phenotypic contrast, inherently introduces selection bias and may exaggerate differences between the groups. Consequently, we were unable to describe a dose–response relationship or establish a specific ferritin threshold, and our results apply primarily to patients at these clinical extremes. Furthermore, while the matched-pair analysis provides additional insights, the relatively small sample size (*n* = 39) limits the statistical power and robustness of the conclusions drawn from this specific sub-group. Additionally, we lacked systematic data on standardized organ dysfunction scores (e.g., SOFA) and ICU-level therapies, which are critical markers of clinical severity. Finally, potential confounders such as underlying liver disease, iron overload syndromes, and transfusion history were not systematically captured and could have influenced the observed ferritin levels.

The economic feasibility and clinical implications of routine ferritin measurement have been addressed in our recent work involving a large cohort of 8759 hospitalized patients. While that study did not constitute a formal cost-effectiveness analysis, it demonstrated that the direct cost of ferritin testing is relatively low, at approximately 1.22 USD per patient. Given the high prevalence of hyperferritinemia in the internal medicine setting, the estimated cost to identify one patient with extreme ferritin levels (>1650 ng/mL)—who carries a significantly elevated risk of in-hospital mortality (29.1%)—was found to be only 36.70 USD. Such a screening strategy may facilitate the early identification of high-risk ‘inflammation-prone’ individuals, potentially guiding more intensive clinical monitoring and prioritizing diagnostic resources for those at the greatest risk of poor outcomes [27].

## 5. Conclusions

We conclude that the addition of ferritin to the routine laboratory work-up upon admission of patients to the department of Internal Medicine with significant inflammation is relevant and conveys additional information regarding patients who are at increased risk of in-hospital mortality. Future prospective studies are necessary in order to find out if this information will be translated into better clinical care and prognosis in this high-risk group of patients.

## Figures and Tables

**Table 1:** Baseline Characteristics of two groups of patients with normoferritinemic versus hyperferritinemic inflammation (full cohort).

Variable	Normoferritinemic Group (*n* = 100)	Hyperferritinemic Group (*n* = 100)	*p* Value
Ferritin (ng/mL)—median (IQR)	88.50 (70.05, 109.85)	2382.10 (1863.07, 4054.00)	
**Demographics**			
Age (years)—median (IQR)	76.3 (60.7, 84.75)	69.2 (60.7, 79.59)	0.068
Male sex—no. (%)	52 (52.0%)	59 (59.0%)	0.319
**Laboratory Values**			
CRP (mg/L)—median (IQR)	176.77 (155.01, 215.68)	207.03 (175.83, 276.23)	<0.001
**Diagnoses on Admission—no. (%)**			
Pneumonia	31 (31.0%)	30 (30.0%)	<0.001 *
Sepsis	5 (5.0%)	28 (28.0%)	
Urinary tract infection	20 (20.0%)	4 (4.0%)	
Cellulitis	17 (17.0%)	5 (5.0%)	
Other infections	14 (14.0%)	14 (14.0%)	
Non-infectious diseases	13 (13.0%)	19 (19.0%)	
**Comorbidities—no. (%)**			
Active Malignancy	8 (8.0%)	55 (55.0%)	<0.001
s/p Cerebrovascular Accident	24 (24.0%)	2 (2.0%)	<0.001
Diabetes Mellitus	43 (43.0%)	25 (25.0%)	0.007
Asthma	6 (6.0%)	0 (0.0%)	0.014
Hypertension	62 (62.0%)	47 (47.0%)	0.033
Chronic Heart Failure	19 (19.0%)	11 (11.0%)	0.113
s/p Malignancy	18 (18.0%)	11 (11.0%)	0.160
s/p Venous Thromboembolism	8 (8.0%)	11 (11.0%)	0.469
Inflammatory Comorbidity	6 (6.0%)	8 (8.0%)	0.579
Ischemic Heart Disease	20 (20.0%)	17 (17.0%)	0.585
Chronic Obstructive Pulmonary Disease	8 (8.0%)	10 (10.0%)	0.621
Dyslipidemia	40 (40.0%)	37 (37.0%)	0.663
Chronic Kidney Disease	12 (12.0%)	13 (13.0%)	0.831
In-hospital mortality—no. (%)	7 (7.0%)	41 (41.0%)	<0.001

* Post hoc analysis revealed statistically significant differences between groups for the diagnoses of sepsis, cellulitis, and urinary tract infections.

**Table 2:** Baseline Characteristics of Patients without active malignancy.

Variable	Normoferritinemic Group (*n* = 92)	Hyperferritinemic Group (*n* = 45)	*p* Value
Ferritin (ng/mL)—median (IQR)	86.65 (69.92, 108.30)	2135.40 (1715.70, 3625.95)	
**Demographics**			
Age (years)—median (IQR)	76.6 (59.9, 84.7)	69.3 (58.2, 83.9)	0.294
Male sex—no. (%)	48 (52.1%)	26 (57.7%)	0.537
**Laboratory Values**			
CRP (mg/L)—median (IQR)	174.69 (155.11, 212.35)	220.94 (191.61, 283.96)	<0.001
**Diagnoses on Admission—no. (%)**			
Pneumonia	29 (31.5%)	17 (37.8%)	0.001 *
Sepsis	5 (5.4%)	13 (28.9%)	
Urinary tract infection	18 (19.6%)	3 (6.7%)	
Cellulitis	16 (17.4%)	4 (8.9%)	
Other infections	13 (14.1%)	7 (15.6%)	
Non-infectious diseases	11 (12.0%)	1 (2.2%)	
**Comorbidities—no. (%)**			
s/p Cerebrovascular Accident	22 (23.9%)	1 (2.2%)	0.001
Diabetes Mellitus	40 (43.5%)	13 (28.9%)	0.100
Asthma	5 (5.4%)	0 (0.0%)	0.172
Hypertension	57 (62.0%)	22 (48.9%)	0.146
Chronic Heart Failure	19 (20.7%)	3 (6.7%)	0.036
s/p Malignancy	16 (17.4%)	4 (8.9%)	0.186
s/p Venous Thromboembolism	8 (8.7%)	4 (8.9%)	>0.999
Inflammatory Comorbidity	6 (6.5%)	4 (8.9%)	0.729
Ischemic Heart Disease	19 (20.7%)	8 (17.8%)	0.691
Chronic Obstructive Pulmonary Disease	8 (8.7%)	6 (13.3%)	0.388
Dyslipidemia	36 (39.1%)	20 (44.4%)	0.552
Chronic Kidney Disease	12 (13.0%)	8 (17.8%)	0.461
In-hospital mortality—no. (%)	6 (6.5%)	14 (31.1%)	<0.001

* Post hoc analysis revealed statistically significant differences between groups for the diagnoses of sepsis and urinary tract infections.

**Table 3:** Causes of death in patients without active malignancies *.

Normoferritinemic Inflammation (*n* = 6) (%)		Hyperferritinemic Inflammation (*n* = 14) (%)	
Pneumonia	3 (50.0%)	Sepsis	7 (50.0%)
Sepsis	1 (16.6%)	Pneumonia	3 (21.4%)
HIV	1 (16.6%)	Endocarditis	2 (14.2%)
Sudden Cardiac Death	1 (16.6%)	Acute Infection	1 (7.1%)
		Ischemic Colitis	1 (7.1%)

* Causes of death are listed in decreasing order of frequency within each group.

**Table 4:** Comparison of matched non-malignant patient pairs according to CRP concentrations.

	Normoferritinemic Inflammation	Hyperferritinemic Inflammation	*p* Value
Number of participants	39	39	
Ferritin (ng/mL)—median (IQR)	90.2 (69.8–110.9)	2135.4 (1725.3–3416.1)	
Age (years)—(Median, IQR)	77.9 (67.8–85.8)	69.3 (57.7–84.4)	0.204
Male sex—no (%)	25 (64.1%)	16 (41.0%)	0.064
CRP (mg/L)—(Median, IQR)	216.7 (190.0–256.5)	219.2 (188.0–255.6)	0.675
In-hospital mortality—no. (%)	1 (2.6%)	10 (25.6%)	0.012

## Data Availability

All original data generated or analyzed in this study are presented in this article. Additional information is available from the corresponding author upon request.

## Abbreviations

The following abbreviations are used in this manuscript:

CRP	C-Reactive Protein
IQR	Interquartile Range
wrCRP	Wide Range C-Reactive Protein
IL	Interleukin
HIV	Human Immunodeficiency Virus
WHO	World Health Organization
CCI	Charlson Comorbidity Index
OR	Odds Ratio
